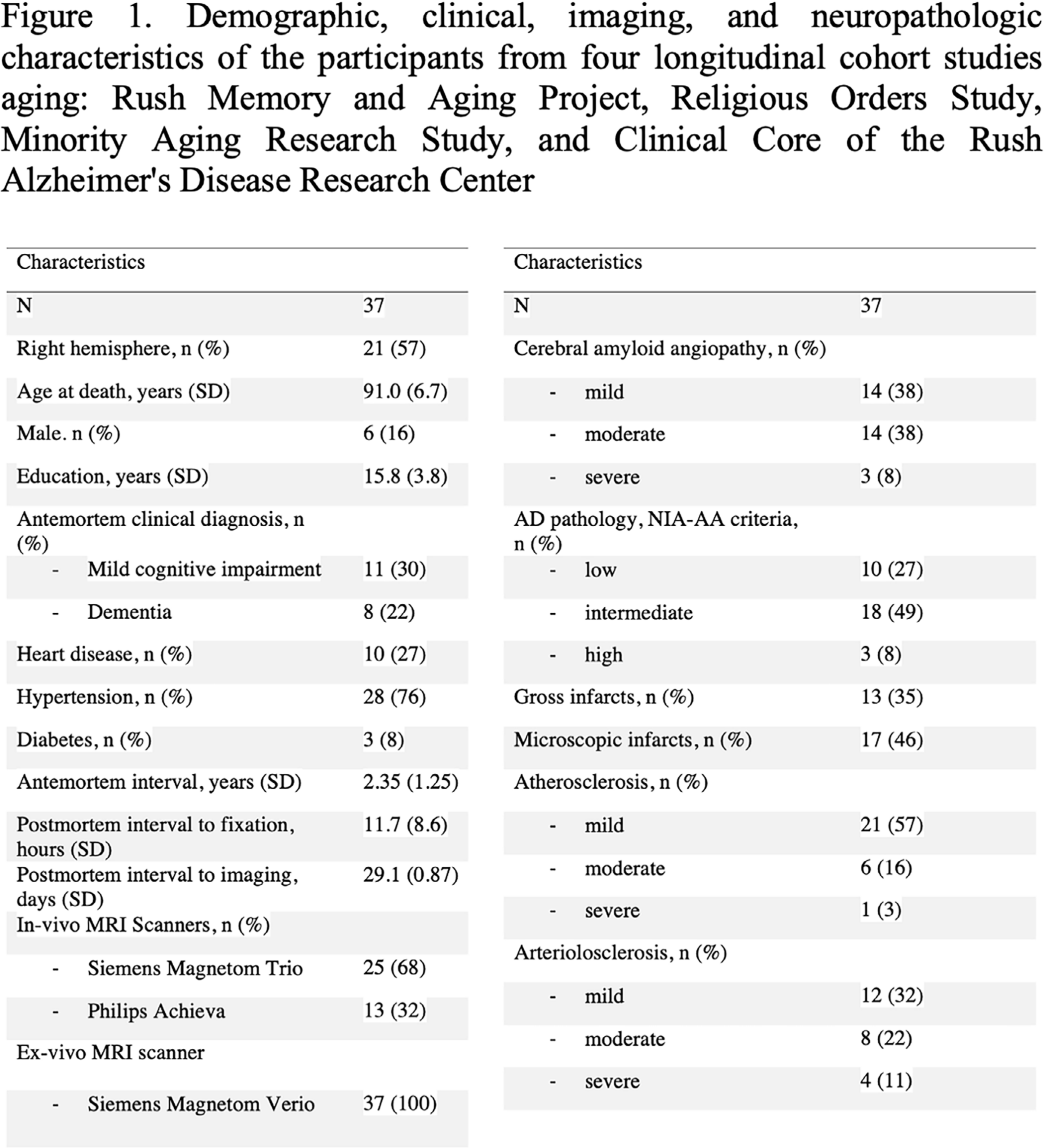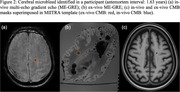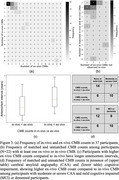# Cerebral microbleeds imaged both in‐vivo and ex‐vivo: A clinical, MRI and neuropathology study in community‐based older adults

**DOI:** 10.1002/alz70855_103441

**Published:** 2025-12-23

**Authors:** Md Tahmid Yasar, Grant Nikseresht, Abdur Raquib Ridwan, Shengwei Zhang, David A. A. Bennett, Julie A Schneider, Konstantinos Arfanakis

**Affiliations:** ^1^ Illinois Institute of Technology, Chicago, IL, USA; ^2^ Rush University Medical Center, Chicago, IL, USA

## Abstract

**Background:**

Cerebral microbleeds (CMBs), seen as small hypointense spots on T2*‐weighted gradient echo MRI, are primarily small brain hemorrhages. CMBs are common in community‐based older adults and are linked to cognitive decline, reduced brain volume, and increased risk of intracerebral hemorrhage, stroke, and mortality. In this study, CMBs that appear in both in‐vivo and ex‐vivo MRI were investigated and the association of these findings with clinical evaluations and neuropathologies were assessed.

**Method:**

The longitudinal study included 37 community‐dwelling older adults who participated in four aging studies (Figure 1). All participants underwent cognitive function testing and brain MRI annually. Whole brain 3D‐T1w MPRAGE (1×1×1mm^3^), and multi‐echo gradient‐echo (ME‐GRE) (0.7×0.7×1.3mm^3^) MRI were acquired in‐vivo using 3T scanners. After death and autopsy, one cerebral hemisphere 3D multi‐echo spin‐echo (ME‐SE) (0.6×0.6×1.5mm^3^) and ME‐GRE (1×1×1mm^3^) of each participant was imaged ex‐vivo with 3T scanners. Following ex‐vivo MRI, all hemispheres underwent neuropathologic assessment including cerebrovascular pathologies like cerebral amyloid angiopathy (CAA), arteriolosclerosis, atherosclerosis, and gross and microscopic infarcts.

CMBs were annotated on the last acquired in‐vivo before death and ex‐vivo ME‐GRE blinded to all pathologic and clinical data. The ex‐vivo CMB mask was first transformed to ME‐SE space, then registered to the MIITRA template. The in‐vivo CMB mask for the corresponding ex‐vivo hemisphere was registered to MPRAGE and subsequently registered to the MIITRA template. The two CMB masks for each participant were then superimposed in MIITRA space to assess chronological changes in CMB characteristics. In‐vivo and ex‐vivo CMBs that overlapped exactly were considered to be the same CMBs and denoted as matched CMBs (Figure 2).

**Result:**

Of the 37 participants, 22 (59%) had at least one CMB in ex‐vivo data, 24 (65%) showed exact match between in‐vivo and ex‐vivo CMBs (Figure 3a, 3b). In all mismatched cases, ex‐vivo CMB counts were higher than in‐vivo. The ex‐vivo CMB counts were higher than in‐vivo in participants with higher antemortem intervals, more cognitive impairment and CAA (Figure 3c, 3d).

**Conclusion:**

This investigation showed that CMBs observed during life persist in the brain and can be detected on ex‐vivo MRI. More CMBs develop over time in individuals with cognitive impairment and CAA.